# Pressure and Composition Effects on a Common Nanoparticle Ligand–Solvent Pair

**DOI:** 10.1021/acs.jpcb.3c06234

**Published:** 2024-01-10

**Authors:** Samuel Salas Sanabria, Lindsey A. Hanson

**Affiliations:** Department of Chemistry, Trinity College, Hartford, Connecticut 06106, United States

## Abstract

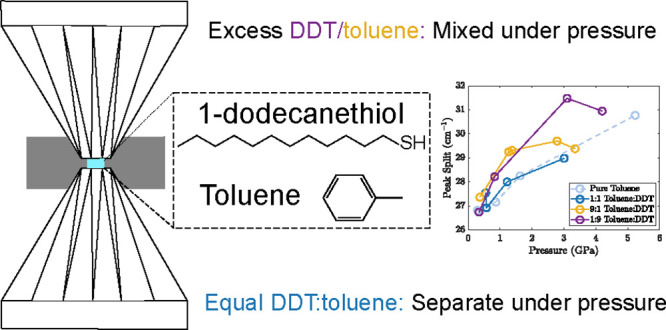

The effect of pressure on the properties of nanoparticles is a growing area of investigation. These measurements are typically performed in a colloidal suspension; however, pressure-induced changes in the interactions between the nanoparticle surface and the solvent are often neglected. Here, we report vibrational spectroscopy of a common nanoparticle ligand, 1-dodecanethiol, and a common solvent, toluene, under pressure. We find that the pressure-induced phase change of the 1-dodecanethiol is altered by the presence of toluene and that change depends on the concentration of the free ligand in the solution. At near-equal concentrations, phase segregation is observed and the dodecanethiol crystallizes independently from the toluene. On the other hand, at unequal concentrations, concerted phase transitions are observed in the dodecanethiol and toluene, and a disordered conformation of dodecanethiol is maintained under much higher pressures. These results shed light on the pressure-induced changes in intermolecular interactions between nanoparticle ligands and solvents, which must be considered in the design of high-pressure investigations of colloidal nanoparticles.

## Introduction

Over the past few decades, colloidal nanoparticles have become materials of great interest in many applications, including electronic, mechanical, optical, and biomedical materials. Pressure is an important variable to consider in the study of the fundamental properties of nanoparticles and the role of size and increased surface area in determining the said properties. Size dependence in high-pressure phase diagrams has been observed in metallic and semiconductor nanocrystals,^1−6^ and similar studies have reported intriguing pressure-dependent optical^7−19^ and mechanical^20−23^ properties of nanocrystals and their assemblies.

In many cases, the nanocrystals of interest are colloidal in nature, capped with organic ligands to passivate the surface and suspend the particles in a solvent, which then serves as the pressure-transmitting medium during compression. In addition to the surface-bound ligand molecules and free solvent, there is an often ill-defined concentration of free ligand molecules in the solution, as well. Depending on the identity of the inorganic core of the nanoparticle and the pressure environment desired, the ligands and solvents vary,^14^ but they can be categorized into hydrophilic ligands like poly(ethylene glycol)^20^ or poly(vinylpyrrolidone),^24^ which are often used to suspend nanocrystals in water or alcohols, and hydrophobic ligands, which are used to suspend nanocrystals in less polar solvents. Some of the most commonly used hydrophobic ligands and solvents are alkylthiols or alkylamines in toluene^8,25,26^ or silicone oil,^16,27^ used as nonhydrostatic pressure transmitting media, as well as paraffin,^28^ pentane/isopentane,^25^ or ethylcyclohexane,^8^ used as quasi-hydrostatic pressure transmitting media. The use of such colloidal nanocrystals allows the particles to be suspended apart from each other in order to eliminate particle–particle interactions during compression. However, with a focus on the behavior of only the nanoparticle under pressure, the effects of the interactions of the organic ligand and solvent molecules have often been neglected.

Due to their industrial importance and astrophysical interest, organic small molecules and their behavior under pressure have been the subject of much research.^29−34^ High pressure allows the effect of intermolecular interactions on conformational and phase changes as well as high-pressure reactions including polymerization to be probed in the absence of thermal effects. In these studies, Raman spectroscopy is often used to monitor conformation changes under pressure.^35−38^ In linear alkanes, with increasing pressure, crystallization is followed by conformational changes and an order–disorder transition that can be followed by changes in intensities of the C–C and C–H stretches associated with the various gauche and trans conformers the molecule.^39^ The ability to monitor conformations and intermolecular interactions, in particular, of alkanes, makes Raman spectroscopy an ideal technique by which to study alkylthiol ligands under pressure.

In order to shed light on potential changes in interactions between common nanoparticle ligands and solvents under pressure, here, we investigate the pressure-dependent interactions between a commonly used ligand, 1-dodecanethiol (DDT), and a common solvent, toluene, by Raman spectroscopy in a diamond anvil cell (DAC). Although much work has been done investigating the intermolecular interactions of alkanes under pressure,^39−42^ to our knowledge none has been reported on either of these molecules. Furthermore, little work, in general, has been reported on the high-pressure behavior of mixtures of organic molecules. Due to the free ligand molecules in a colloidal sample, the solution is likely to be a mixture of ligand and solvent as well as suspended nanoparticles. Therefore, here, we investigate not only the phase behavior of each pure component but also the effect on concentration as well. We observe pressure and composition dependence of the molecular conformation of DDT that indicates the ligand–solvent interaction is important to consider in the design of high-pressure experiments on colloidal nanoparticles.

## Methods

Samples were prepared in 1:9, 9:1, and 1:1 proportion-by-volume mixtures of toluene (Fisher, Certified ACS) and 1-dodecanethiol (Acros, 98%). Pure samples of each compound were similarly studied. High-pressure compression was performed in a Bx90 Deutsches Elektronen-Synchrotron (DESY), Hamburg, DAC, with all measurements performed at room temperature. 500 μm diameter type IIa diamonds (Technodiamant USA) were used in the DAC. Spring steel gaskets were prepared by indentation with the diamonds to a final thickness of 80 μm. A 150 μm hole was drilled in the center of each gasket indentation by electric discharge milling (Hylozoic Products, Seattle, WA). Ruby powder (Almax easyLab) was placed in the sample chamber along with the liquid sample, which also served as the pressure-transmitting medium. The sample chamber was inspected by bright-field imaging with a Leica dmi8 microscope equipped with a 10× objective (NA = 0.28, Mitutoyo). A Horiba XploRA confocal Raman microscope was calibrated using a silicon standard and operated with a 532 nm laser with a power between 20 and 25 mW. The DAC was integrated into the Raman spectrometer and data was collected through a 10× objective (NA = 0.25, Olympus). A 500 μm aperture and 100 μm slits were used, and for each acquisition, 20 spectra were averaged. Laser power and exposure time were modified accordingly to maximize the signal-to-noise ratio for each sample. A 600 g/mm grating was used for a lower resolution, higher bandwidth spectrum, while a 2400 g/mm grating was performed for higher resolution spectra. Ruby fluorescence was collected using an 1800 g/mm grating centered at 690 nm, and the position of the R2 line was used to calibrate the pressure.^43^ The uncertainty in the calculated pressure was estimated to be ±0.1 GPa by repeated measurements. Spectra were fit to a sum of Gaussian functions using a least-squares Levenberg–Marquardt algorithm, and uncertainties in peak positions and widths were evaluated from the covariance of the fit.

## Results and Discussion

**Figure 1:**
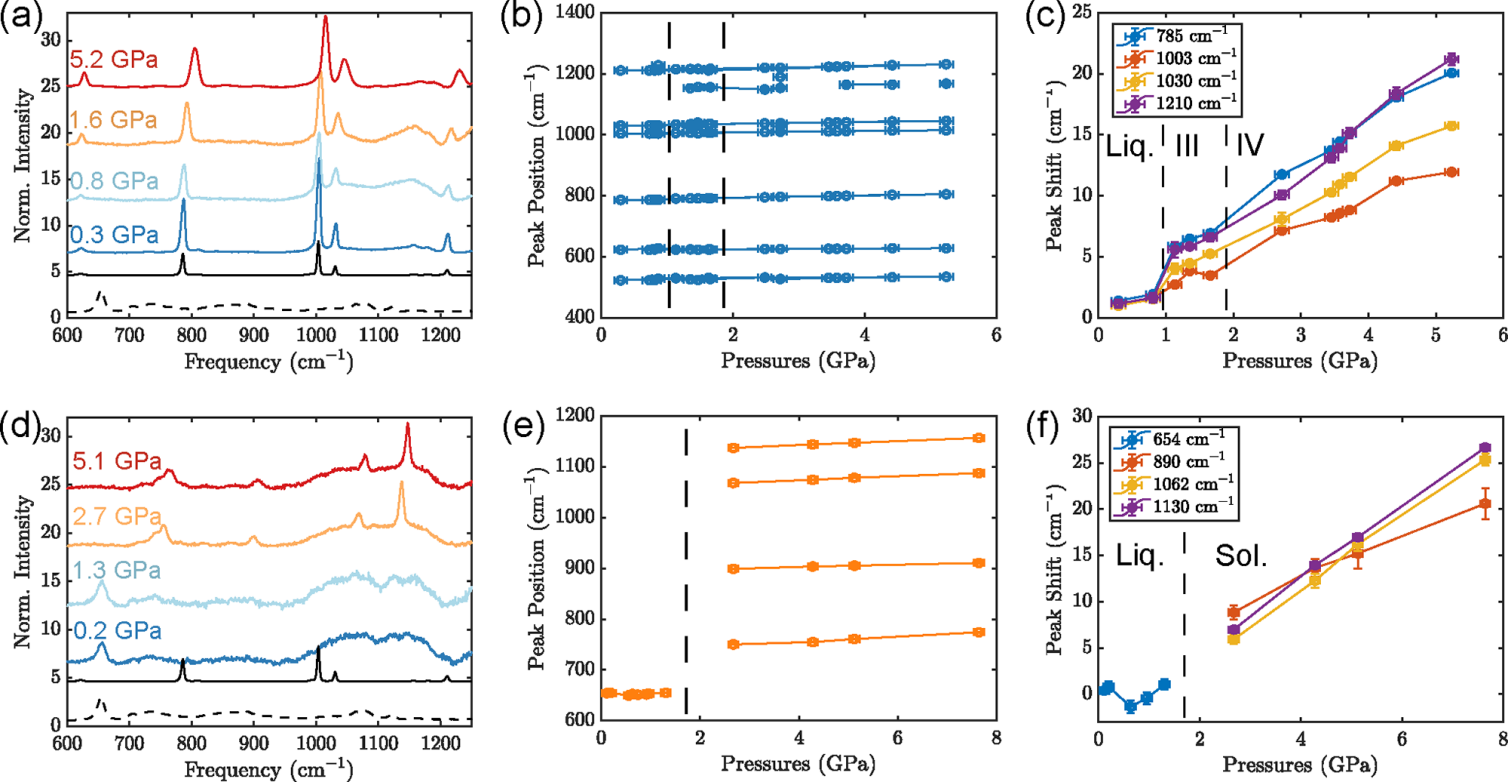
Behavior of pure toluene (a–c) and pure 1-dodecanethiol (d–f) under pressure. (a) Raman spectra of pure toluene with increasing pressure. (b) Raman peak positions of pure toluene vs pressure. (c) Raman peak shifts relative to the ambient pressure of pure toluene vs pressure. (d) Raman spectra of pure 1-dodecanethiol with increasing pressure. (e) Raman peak positions of pure 1-dodecanethiol vs pressure. (f) Raman peak shifts relative to the ambient pressure of pure 1-dodecanethiol versus pressure. In (a,d), the ambient pressure spectrum of pure toluene (solid black) and pure 1-dodecanethiol (dotted black) are shown below for reference. In (b,c,e,f), phase transitions are indicated with vertical dotted lines.

To begin, we characterized the behavior of pure dodecanethiol and pure toluene under pressure independently. Figure 1 shows selected Raman spectra of each neat substance in a region of 500 to 1250 cm^–1^ at various pressures. Upon pressurization, changes in peak positions, widths, and relative intensities can be observed, which gives insight into the molecular conformations and phase changes of both substances. The spectra of each pure liquid at ambient pressure are shown below the spectra under pressure for reference. The ambient pressure spectra of the pure substances and mixtures are also shown in Supporting Information Figure S1 with the peak assignments used hereafter.

In pure toluene (Figure 1a), ring breathing and C–H bending vibrations are observed at all measured pressures, up to 5.2 GPa, and no appearance of new peaks or splitting is observed (Figure 1b). All peaks blue-shift to higher frequencies with increasing pressure (Figure 1c), and a liquid–solid phase transition at 0.9 GPa is evident as a discontinuity in the shift with pressure, in keeping with the high-pressure solidification of toluene reported in the literature.^44,45^ Changes in peak width are also seen at the liquid–solid transition at 0.9 GPa and again at 1.9 GPa, in accordance with a solid–solid transition also reported in the literature.^45^ In particular, the peak width increases markedly after the phase transition at 1.9 GPa (Supporting Information Figure S2).

In pure dodecanethiol (Figure 1d), more dramatic changes are seen in the vibrations present near ambient pressure compared to those at pressures of several GPa. In particular, at low pressures primarily vibrations associated with gauche conformers, both in the C–S stretch [ν(C–S)_G_] at 655 cm^–1^ and the broad mixture of C–C stretches [ν(C–C)_G_] between 1000 and 1200 cm^–1^, are in accordance with previous reports of dodecanethiol in the bulk liquid form.^46,47^ A phase transition occurs between 1.3 and 2.7 GPa, which is marked by a dramatic change in the vibrations present (Figure 1e). Above the phase transition, the trans conformer of the C–S stretch [ν(C–S)_T_] is the only one observed, with a peak at 735 cm^–1^ that overlaps with that of the solid CH_2_ rock at 749 cm^–1^. No intensity is observed in the C–S gauche region at around 655 cm^–1^. Above the same transition, trans C–C stretching [ν(C–C)_T_] peaks are seen at 1062 and 1132 cm^–1^. Both ν(C–S)_T_ and ν(C–C)_T_ persist up to the highest pressures measured, close to 8 GPa. A small peak at 1084 cm^–1^ is present at intermediate pressures above the phase transition that indicates the presence of C–C gauche conformers, which disappear entirely at higher pressures (Supporting Information Figure S3). This indicates the formation of an intermediate rotator phase of the alkane before crystallization, which has been observed in other linear alkanes under pressure.^39^ However, no rotator phase is seen in *n*-dodecane,^32^ so the presence of the thiol clearly alters the phase diagram of 1-dodecanethiol.

In addition to the appearance and disappearance of peaks that indicate the phase changes of 1-dodecanethiol, one can identify the phase change by changes in the peak frequencies and widths as well.^40^ Upon the liquid–solid phase transition, not only does the set of vibrational modes present change but also the shift with pressure of the solid modes is higher than that of the liquid modes (Figure 1f). A marked narrowing is also seen in the majority of the vibrations associated with DDT, in particular, the C–C stretches (Supporting Information Figure S2). Above the phase transition, most vibrations broaden as is commonly seen with increased intermolecular interactions.

**Figure 2:**
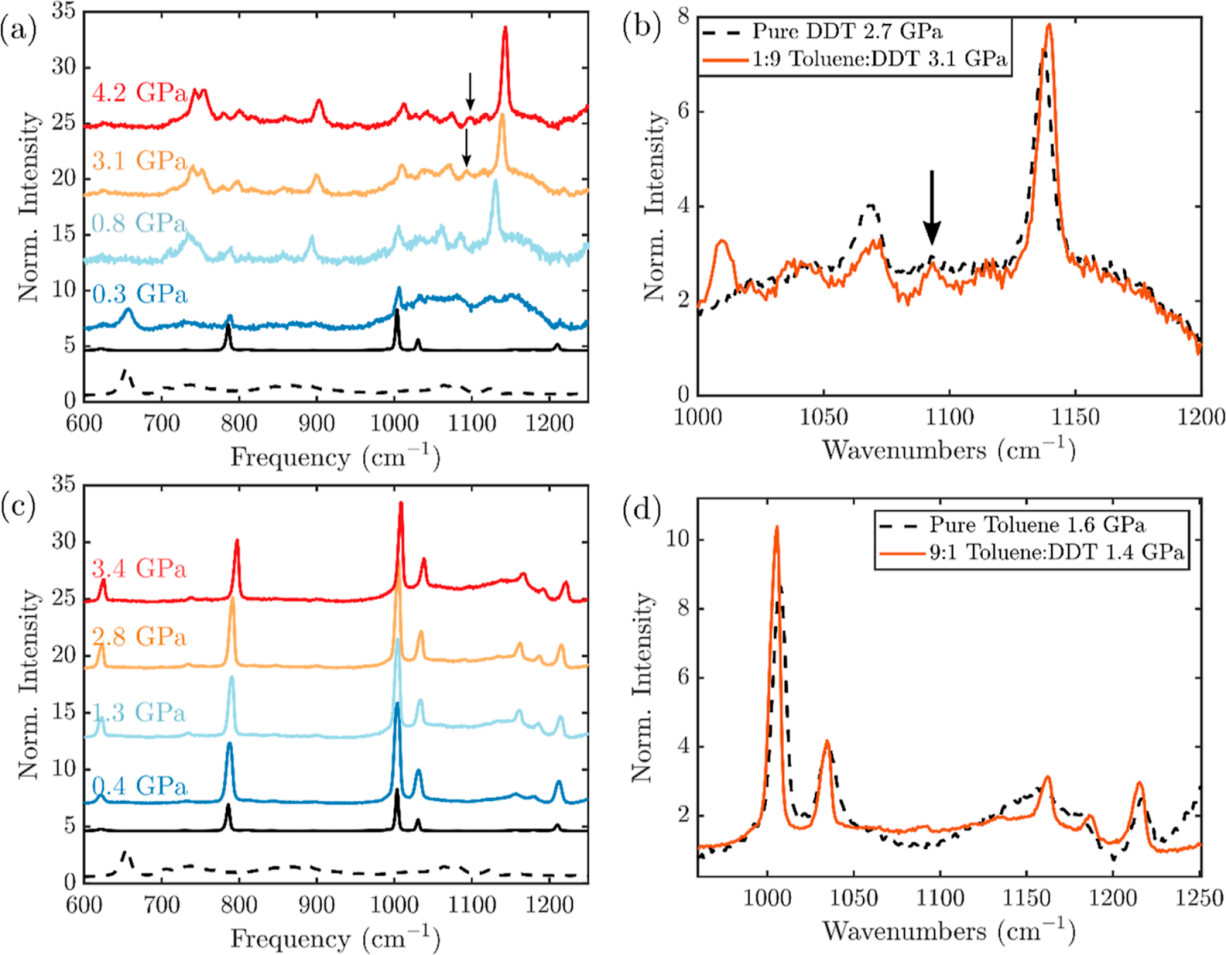
Behavior of unequal mixtures of toluene and 1-dodecanethiol under pressure. (a) Raman spectra of a 9:1 DDT/toluene mixture with increasing pressure. Ambient pressure spectra of pure toluene (solid black) and pure 1-dodecanethiol (dotted black) are shown below for reference. (b) Comparison of Raman spectra of 9:1 DDT/toluene mixture (solid red) and pure toluene (dotted black) at similar pressures. (c) Raman spectra of a 9:1 toluene/DDT mixture with increasing pressure. Ambient pressure spectra of pure toluene (solid black) and pure 1-dodecanethiol (dotted black) are shown below for reference. (d) Comparison of Raman spectra of 9:1 toluene/DDT mixture (solid red) and pure 1-dodecanethiol (dotted black) at similar pressures. Arrows in parts (c,d) indicate C–C stretch modes associated with gauche conformers, which are present at increased proportions in the mixture.

With the behavior of pure dodecanethiol and toluene under pressure as a guide, we can now examine the behavior of mixtures of the two molecules. We began with mixtures of unequal compositions, with 10% by volume either dodecanethiol in toluene or toluene in dodecanethiol. First, we examined a mixture prepared at ambient pressure with a 9:1 ratio of dodecanethiol to toluene by volume (Figure 2). The Raman spectrum of the mixture at ambient pressure (Supporting Information Figure S1) shows that each peak can be assigned to the corresponding vibration in the pure liquid, and no shifts are observed due to the formation of the mixture. The spectrum can thus be produced as a linear combination of the two pure liquids. This is reasonable as no strong interactions are expected between these two molecules. However, in the Raman spectra of the mixture under pressure (Figure 2a), differing behavior is observed in the mixture compared to the neat substances. To begin with, a phase transition is observed below 0.8 GPa by both optical microscopy and the gauche–trans conformer transition of the C–S stretch of 1-dodecanethiol occurs. This transition occurs at much lower pressures than in pure DDT (see the Supporting Information Figure S4 for a direct comparison of the mixture at 0.8 GPa, which clearly shows a predominantly trans conformation, and pure DDT at 1.3 GPa, which persists in the gauche conformation). At the same time, however, a clear peak attributable to the gauche C–C stretch at 1080 cm^–1^ appears at 0.8 GPa and persists at levels as high as 4.2 GPa. This peak is much more prominent in the compressed mixture than in pure dodecanethiol at similar pressures (Figure 2b). Both gauche and trans methylene rocking vibrations are also clear as high as 4.2 GPa. (The presence and peak frequency of each vibrational mode of the mixture are shown in Supporting Information Figure S4). With these spectra, we can see that while the C–S bonds convert completely to a trans conformer at lower pressures in the presence of even a small amount of toluene, the disordered rotator phase of the alkane chain is stabilized to even higher pressures.

With regard to the stability of the 9:1 DDT/toluene mixture under pressure, in addition to a clear effect of the mixture on the conformation of DDT over a wide range of pressures, we also observe the effects of the mixture on both peak shifts and peak widths of toluene and DDT under pressure. The effect of pressure on the vibrations assigned to toluene is changed by the presence of the DDT, exhibiting an initial red shift with increasing pressure that is not seen in pure toluene (Supporting Information Figure S5). This red shift coincides with the apparent liquid–solid transition of the mixture. Subsequently, that red shift relative to pure toluene is maintained as pressure increases, indicating continued interaction with the DDT. Additionally, the conformational change of the DDT coincides with this red shift in the toluene (Supporting Information Figure S5), which supports the idea that the depression of the phase transition pressure of the DDT is due to interactions with toluene. Furthermore, the changes in peak widths of both molecules with pressure are altered in the mixture, and the observed changes coincide with each other (Supporting Information Figure S5). These observations together, along with the alteration of the conformation of the DDT alkane chain, confirm that the two components stay mixed under pressure, even upon solidification.

Similar evidence of the stability of the mixture is seen with toluene in excess. With 10% dodecanethiol in toluene, the spectra are dominated by the vibrations attributed to toluene (Figure 2c). The presence and peak frequency of each vibrational mode of the mixture are shown in Supporting Information Figure S6, although only toluene vibrations are detected. Comparison of the spectrum of the mixture to that of pure toluene at similar pressures (Figure 2d) reveals that the spectrum of the mixture, although exhibiting the same vibrational modes, has many more distinct peaks for said modes under pressure. This is due to a narrowing with pressure up to 3 GPa, in spite of the observation of solidification by optical microscopy rather than the broadening seen in pure toluene (Supporting Information Figure S6). Concomitant with the peak narrowing, a blue shift of several modes relative to their respective behavior in pure toluene is seen (Supporting Information Figure S6), similar to the effect of the interaction with DDT seen in 9:1 DDT/toluene. These changes in the vibrations of 9:1 toluene/DDT under pressure indicate that the two molecules are interacting and thus remain mixed throughout.

**Figure 3:**
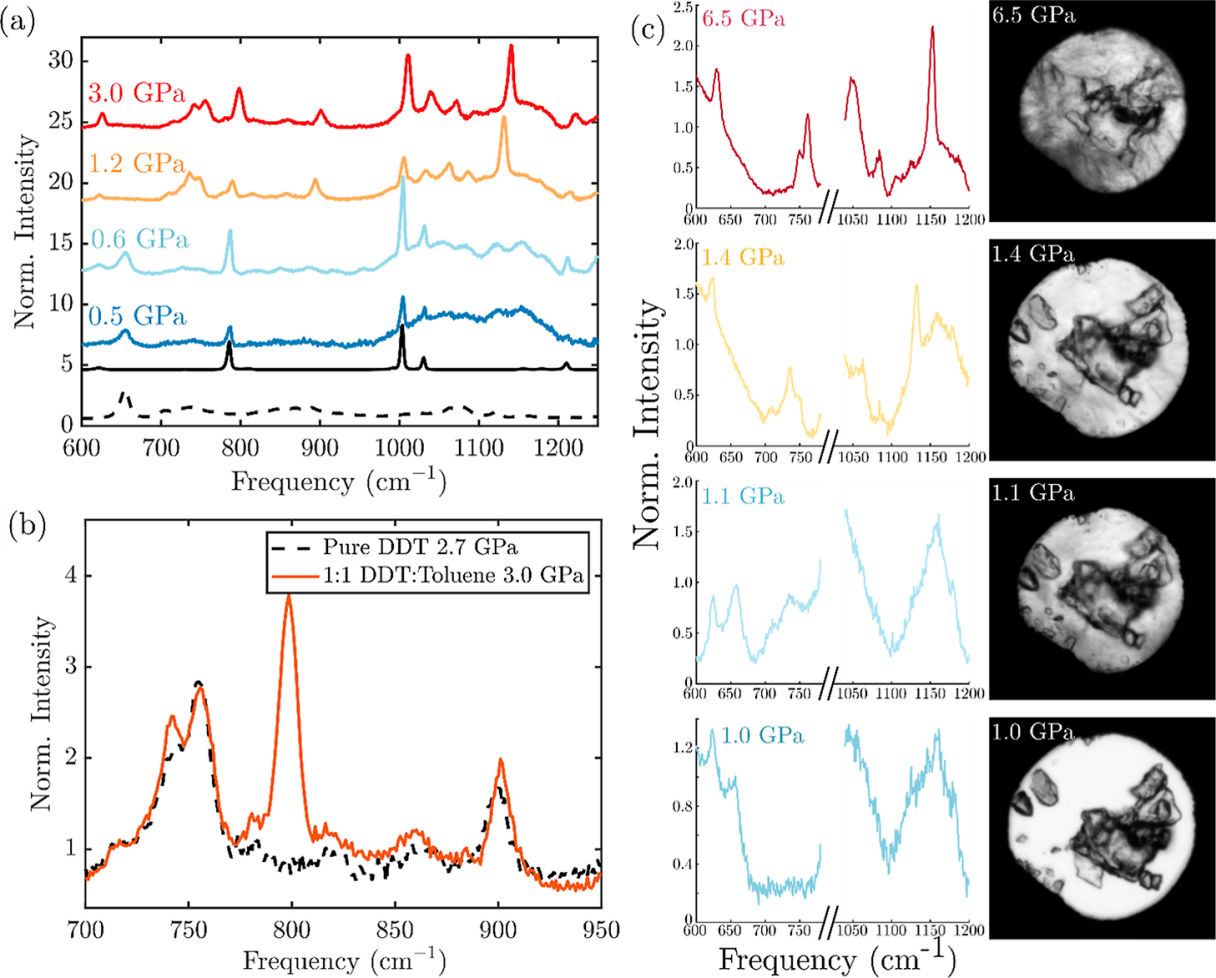
Behavior of a 1:1 mixture of toluene and 1-dodecanethiol under pressure. (a) Raman spectra of the mixture with increasing pressure. Ambient pressure spectra of pure toluene (solid black) and pure 1-dodecanethiol (dotted black) are shown below for reference. (b) Comparison of the ν(C–S) and ρ(CH_2_) regions of the Raman spectra of the mixture (solid red) and pure 1-dodecanethiol (dotted black) at similar pressures. (c) Raman spectra of the mixture (left) with corresponding bright-field microscopy images. At low pressure, all objects seen are ruby grains, and the chamber between them is clear. At high pressure crystalline boundaries appear between the ruby pieces, and the resulting increase in light scattering darkens the entire chamber. Note that at 1.1 GPa, the chamber appears frozen, but the Raman spectrum appears liquid-like.

Lastly, we examined a mixture prepared with equal volumes of dodecanethiol and toluene at ambient pressure (Figure 3). In this case, the behavior of the mixture approaches that of the pure toluene and dodecanethiol, as if they transition independently. The Raman spectra of the 1:1 toluene/DDT mixture under pressure are shown in Figure 3a. The transition from gauche to trans conformers is seen at pressures higher than those of the phase transitions seen in the other mixtures, closer to that seen in pure DDT, with the gauche to trans transition of the C–S stretch occurring around 1.2 GPa. Comparison of the spectrum of the mixture to that of pure DDT at a similar pressure near 3 GPa reveals similar peak positions and intensities in both the ν(C–S) (Figure 3b) and ν(C–C) regions (Supporting Information Figure S7). However, the ν(C–S)_T_ peak is more prominent and better resolved from the ρ(CH_2_) peak than in pure DDT. The trans conformers in the alkane chain are prominent by 1.2 GPa, although they coexist with gauche conformers in a rotator phase at that pressure. The intensity of gauche C–C stretches decreases by 3 GPa, again similar to the decrease seen in pure DDT (Supporting Information Figure S7). The presence and peak frequency of each vibrational mode of the mixture are shown in Supporting Information Figure S7.

The similarity to the behavior of each pure component is further supported upon examination of the peak shifts of each vibration versus pressure. Both below and above the freezing pressure, both the vibrations assigned to toluene and those assigned to DDT exhibit similar shifts from ambient pressure to those of the pure substances (Supporting Information Figure S8). Furthermore, the peak widths of each component also behave as pure substances (Supporting Information Figure S7). These observations point toward the independent behavior of the two components.

More clearly, we still observe independent phase transitions of the toluene and DDT by contrasting the appearance of the sample chamber by optical microscopy with the conformation changes measured by Raman spectroscopy (Figure 3c). When first loaded in the DAC, the mixture appears uniform, with only ruby grains visible in the homogeneous sample chamber. It should be noted that there are many ruby grains visible, but that the space between them, occupied only by the toluene–DDT mixture, is clear of any structure. The liquidity of the sample at this pressure was also confirmed by the observation of Brownian motion of the smaller ruby pieces. With increasing pressure, but before the appearance of large crystals, puncta appear that coincide with increased intensity in the trans conformer C–S stretch of the DDT (Supporting Information Figure S9). Above 1.4 GPa, the chamber visibly freezes into large grains, and the DDT is predominantly in the trans conformers observed with both the C–S and C–C stretches. Upon subsequent depressurization, however, the conformational change of the DDT to the liquid state precedes the apparent melting of the sample chamber. Thus, the DDT undergoes a solid–liquid phase transition independently of toluene, which then melts upon further decreasing pressure. This independent phase transition behavior further supports the interpretation that the mixture with equal parts toluene and DDT, though miscible at ambient pressure, phase segregates at higher pressure.

**Figure 4:**
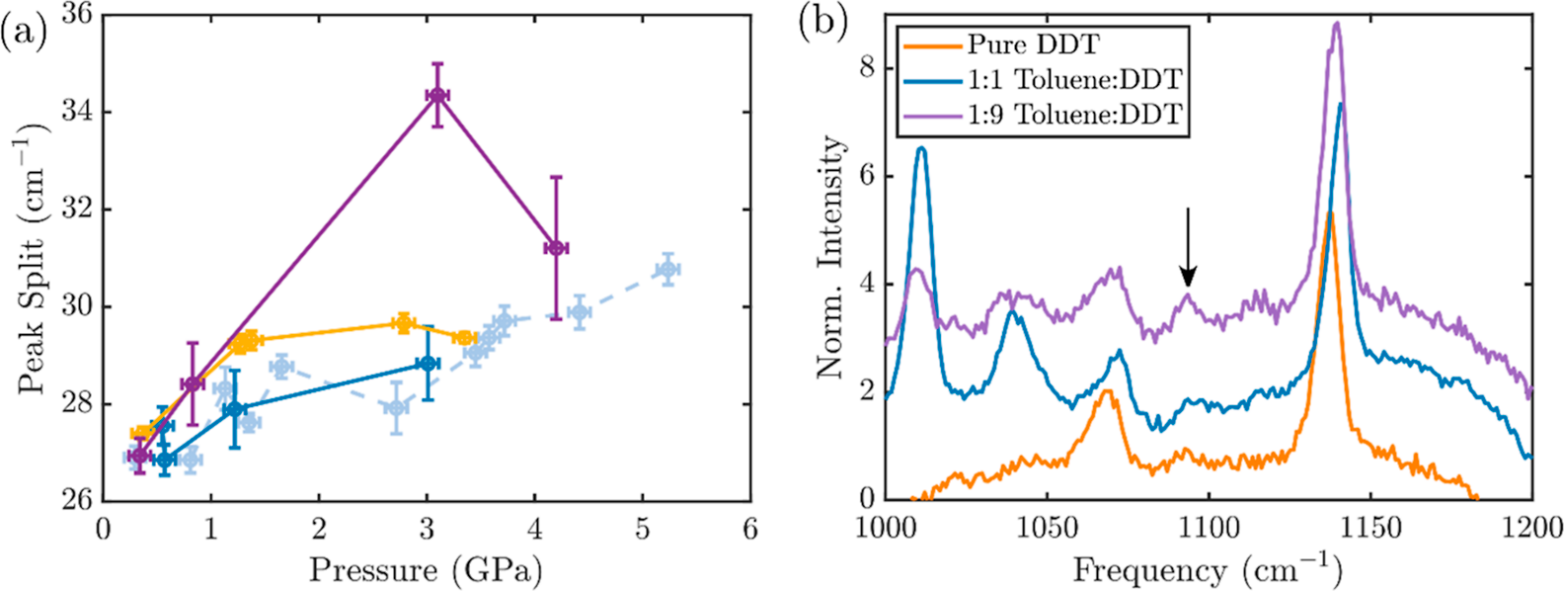
Comparison of mixtures to pure toluene and DDT. (a) Split between 1003 and 1030 cm^–1^ modes of toluene versus pressure. (b) C–C stretch of Raman spectra at similar pressures of pure DDT (2.7 GPa), 1:1 toluene/DDT (3.0 GPa), and 1:9 toluene/DDT (3.1 GPa).

The contrast between the behavior of the mixtures at various compositions is particularly evident by direct comparisons. First, in pure toluene the frequency difference between the symmetric ring stretch at 1003 cm^–1^ and the in-plane C–H bending mode at 1030 cm^–1^ increases with pressure (Figure 4a), revealing the increased interactions of the aromatic hydrogens with the neighboring toluene molecules, as has been observed previously.^45^ In the 1:1 mixture, this split increases with pressure to the same extent as that in pure toluene. However, in the 1:9 and 9:1 toluene/DDT mixtures, the difference between these two modes increases more quickly with pressure, indicating a molecular environment that differentially impacts the in-plane C–H bending of toluene in a manner distinct from that of pure toluene. In another comparison, a close examination of the C–C stretch of pure DDT, the 1:1 toluene/DDT mixture, and the 1:9 toluene/DDT mixture at similar pressures near 3 GPa (Figure 4b) reveals again the similarity of the 1:1 mixture to pure DDT, while the 1:9 mixture shows much more intensity in the C–C stretch of the gauche conformation. This indicates disruption of molecular packing of the alkane chains, which leads to increased rotational disorder, which is again distinct from that observed in pure DDT under pressure. The combination of these two observations—the stiffening of the in-plane C–H bend of toluene and the increased rotational disorder of the DDT—indicates increased interactions between toluene and DDT in the unequal mixtures under pressure. While the specific effects observed likely indicate the intercalation of toluene between the alkane chains of DDT in the mixture, determination of the exact nature of the interaction is of great interest and will be the subject of future work.

## Conclusions

These observations present an interesting observation of the phase behavior of a mixture of 1-dodecanethiol and toluene. By careful examination of the Raman spectra of both components under pressure, we are able to establish that very unequal mixtures of toluene and DDT remain stable as a fluid mixture under pressure and solidify as a solid mixture. However, at equal proportions, high-pressure immiscibility is observed, as confirmed by independent crystallization of each molecule separate from the other. This trend is consistent with prior observations of the phase diagrams of other fluids under pressure,^48,49^ in which mixtures with positive excess molar volumes can exhibit high-pressure immiscibility. While no literature on the excess molar volume of mixtures of 1-dodecanethiol and toluene could be found, previous measurements of binary mixtures of dodecane and toluene have the largest excess molar volume at near equal proportions,^50^ while at very unequal proportions, the excess molar volume diminishes greatly, which would reduce the driving force for phase segregation under pressure. These prior observations are in agreement with our findings here, in which the uniform mixture is maintained through to solidification in unequal mixtures, but pressure-induced phase segregation prior to solidification is seen in near-equal proportions. To the best of our knowledge, this is the first report of high-pressure phase segregation of a nonpolar mixture. Furthermore, this knowledge of the phase stability of this particular binary system is important for the design of high-pressure studies of colloidal nanoparticles, in which control over the concentration of free 1-dodecanethiol may be crucial for the maintenance of colloidal stability in toluene. Future studies will extend to include dodecanethiol-capped nanoparticles to explore the effect of phase segregation of a mixed solvent on the colloidal stability of the nanoparticles under pressure.

## Supporting Information Available

Additional characterization results and methods, additional Raman spectra, optical microscopy images, and analysis (PDF)
