# Supplementary material for: Pressure and Composition Effects on a Common Nanoparticle Ligand–Solvent Pair
Source: J Phys Chem B. 2024 Jan 10;128(3):841–8. doi: 10.1021/acs.jpcb.3c06234 (PMC10823465; doi:10.1021/acs.jpcb.3c06234)
Supplement: Supplementary file 1 — jp3c06234_si_001.pdf [file jp3c06234_si_001.pdf]

Pressure and Composition Effects on a Common Nanoparticle Ligand-Solvent Pair

Supplementary Information

Samuel Salas Sanabria[1], Lindsey A. Hanson[1]*

[1]Department of Chemistry, Trinity College, Hartford, CT 06106, USA

[*] Corresponding Author: lindsey.hanson@trincoll.edu

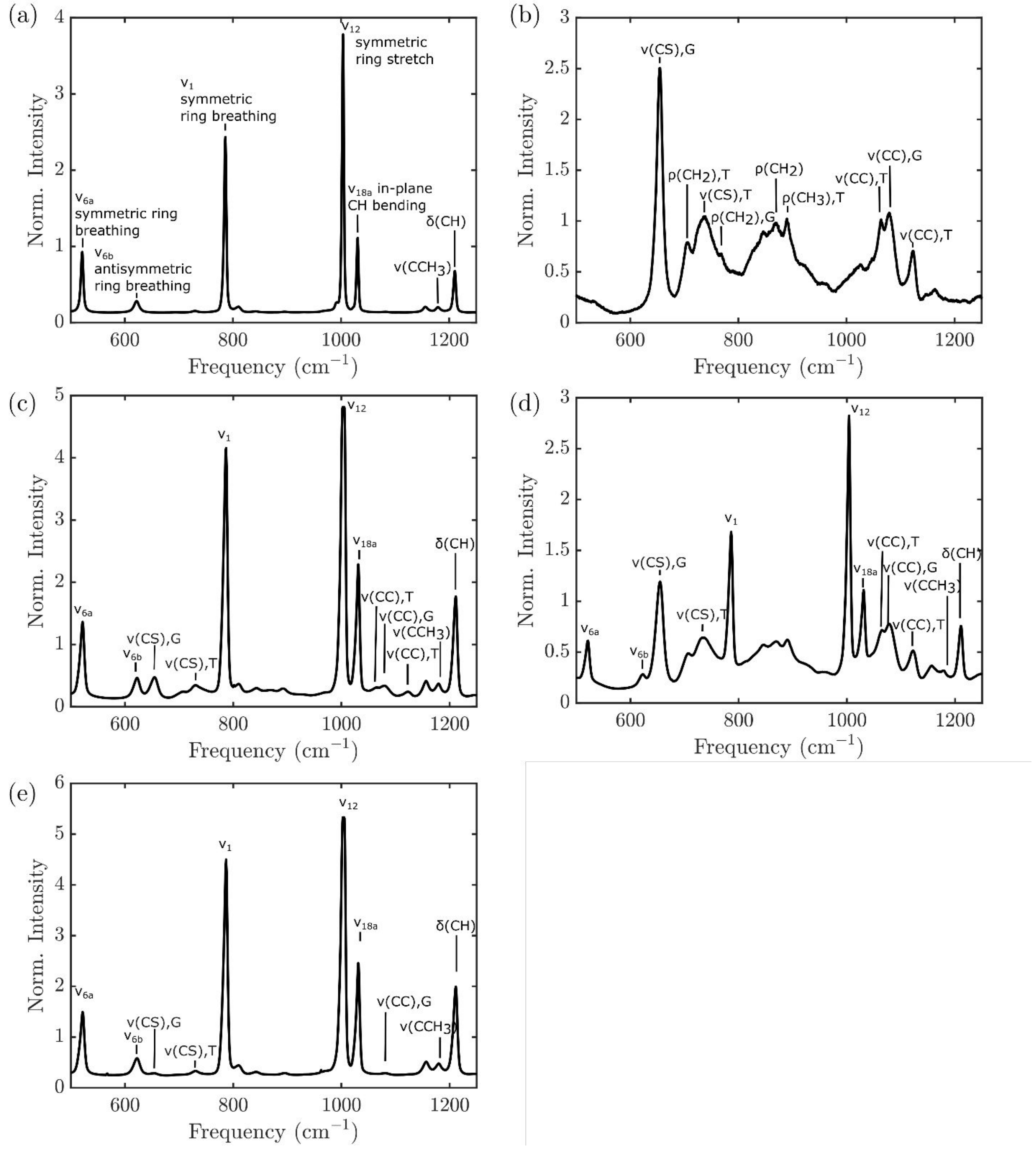

Figure S1. Raman spectra at ambient pressure of (a) pure toluene, (b) pure 1-dodecanethiol, (c) 1:1 toluene:DDT, (d) 9:1 DDT:toluene and (e) 9:1 toluene:DDT. In vibrational mode assignments, ν represents stretching, δ bending and ρ rocking. Assignments are made according to prior literature[1–3].

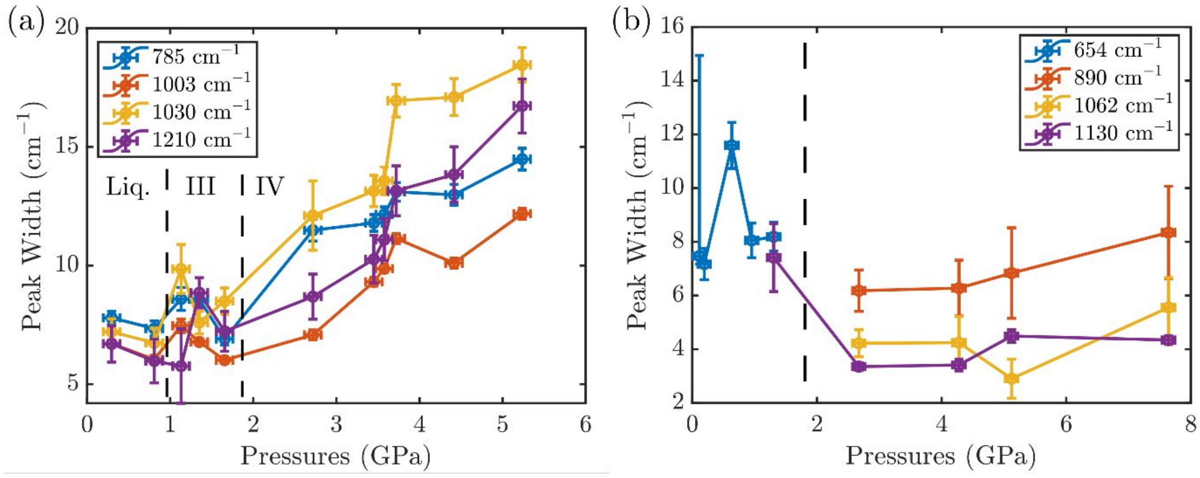

Figure S2. Widths of Raman peaks of pure toluene (a) and pure 1-dodecanethiol (b) versus pressure.

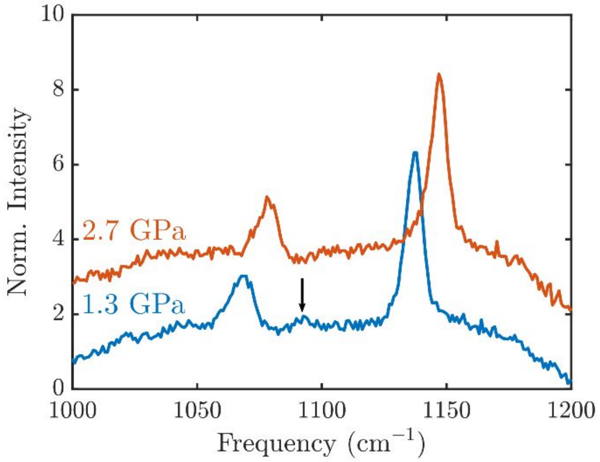

Figure S3. Raman spectra of pure 1-dodecanethiol thiol at 1.3 GPa and 2.7 GPa. The two most prominent peaks at both pressures are the trans C-C stretches at 1064 and 1130 cm^{-1}. The arrow indicates a small peak assigned to the gauche C-C stretch at 1080 cm^{-1}.

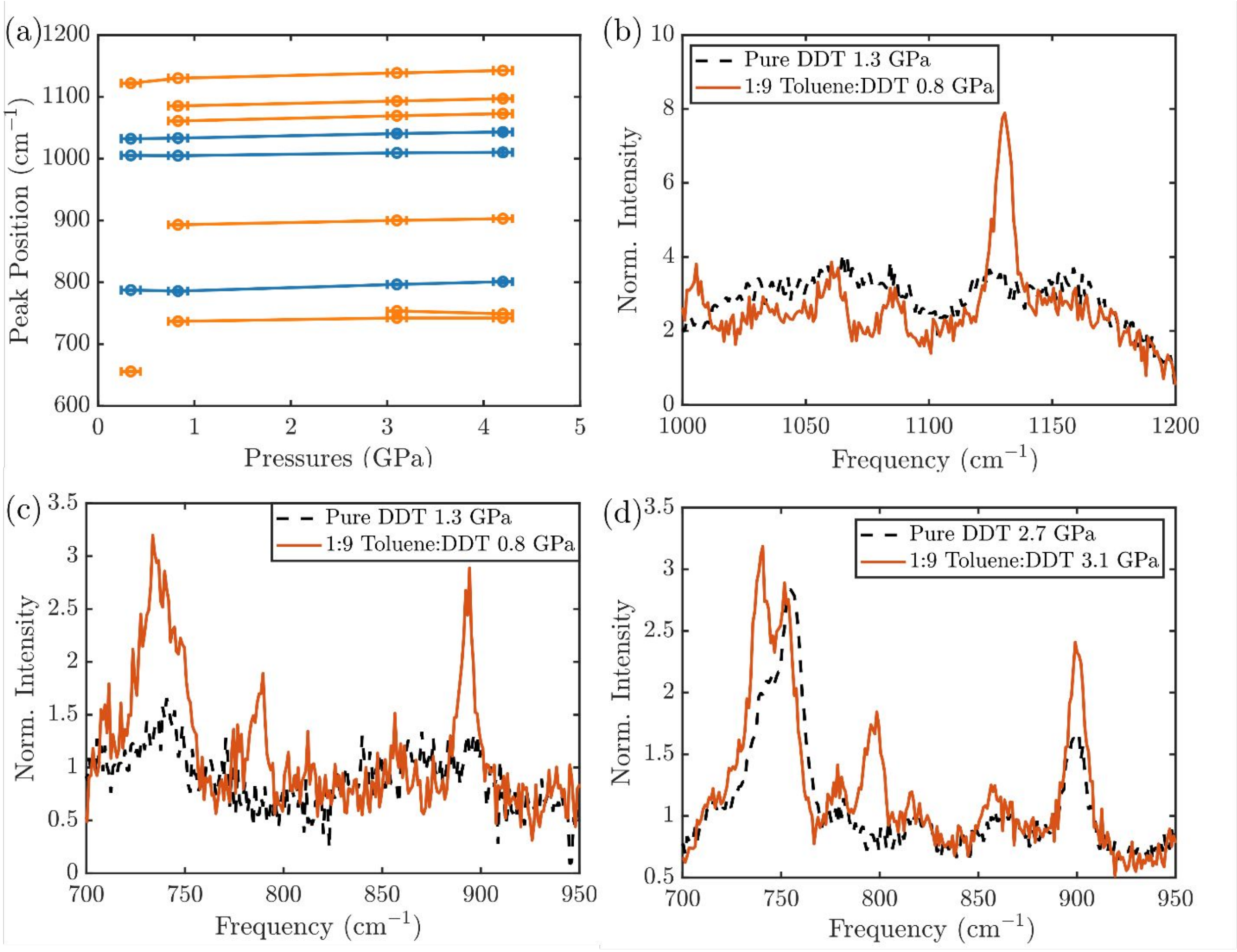

Figure S4. Characterization of Raman spectroscopy of 1:9 toluene:DDT mixture under pressure. (a) Peak positions of identified Raman vibrational modes versus pressure. Peaks identified as from DDT shown in orange, toluene in blue. (b-d) Comparison of Raman spectra of pure DDT (dashed black) and 1:9 toluene:DDT (solid red) at similar pressures. Near 1 GPa, both the (b) C-C stretch region and (c) C-S stretch region show solid-like DDT at much lower pressures in the mixture. (d) The C-S stretch region near 3 GPa shows more intensity of the C-S trans stretch and methyl rock than pure DDT.

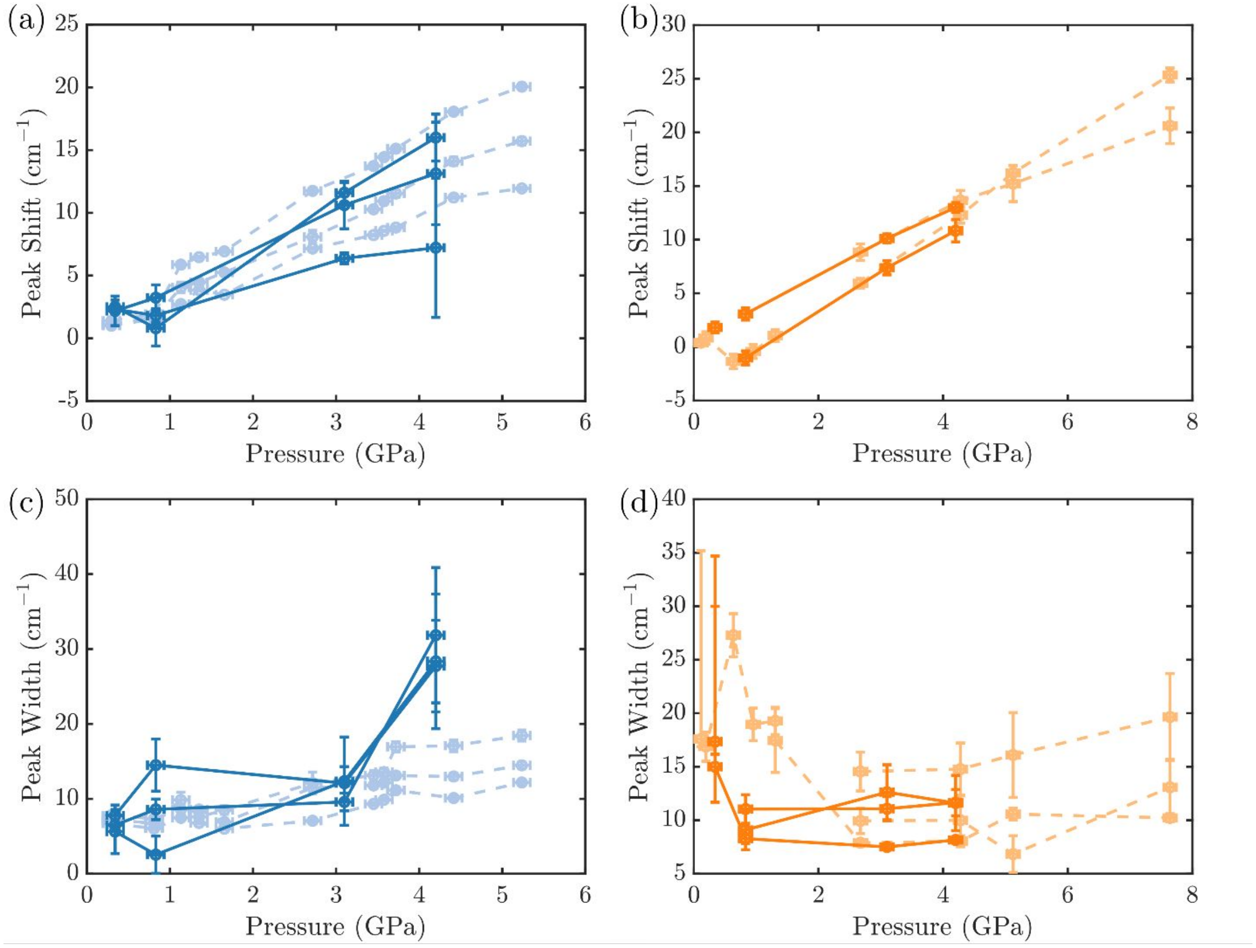

Figure S5. Characterization of Raman spectroscopy of 1:9 toluene:DDT mixture under pressure. (a) Raman peak shift of toluene vibrational modes in mixture (solid lines) compared to pure toluene (dashed lines) versus pressure. (b) Raman peak shift of 1-dodecanethiol vibrational modes in mixture (solid lines) compared to pure 1-dodecanethiol (dashed lines) versus pressure. (c) Widths of Raman peaks of toluene in the 1:9 toluene:DDT mixture versus pressure. (d) Widths of Raman peaks of DDT in the mixture versus pressure.

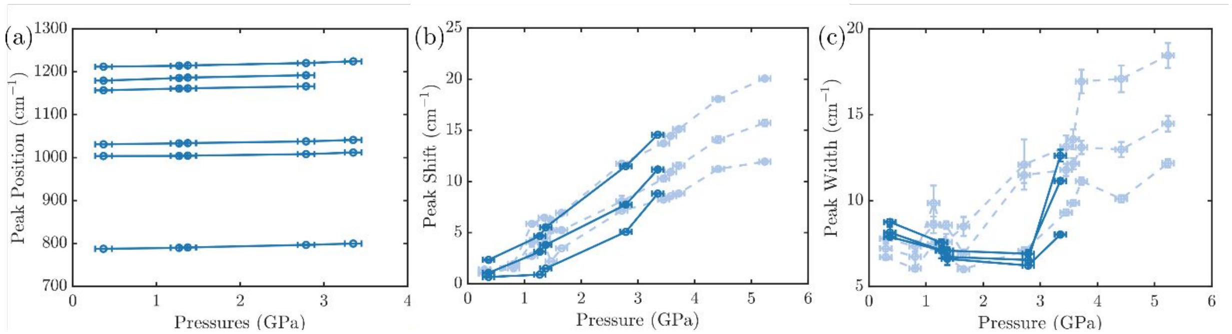

Figure S6. (a) Peak center frequencies of 9:1 toluene:DDT versus pressure. All modes are identified as from toluene. (b) Raman peak shift of toluene in mixture (solid lines) compared to pure toluene (dashed lines) versus pressure. (c) Raman peak width of toluene in mixture (solid lines) to pure toluene (dashed lines) versus pressure.

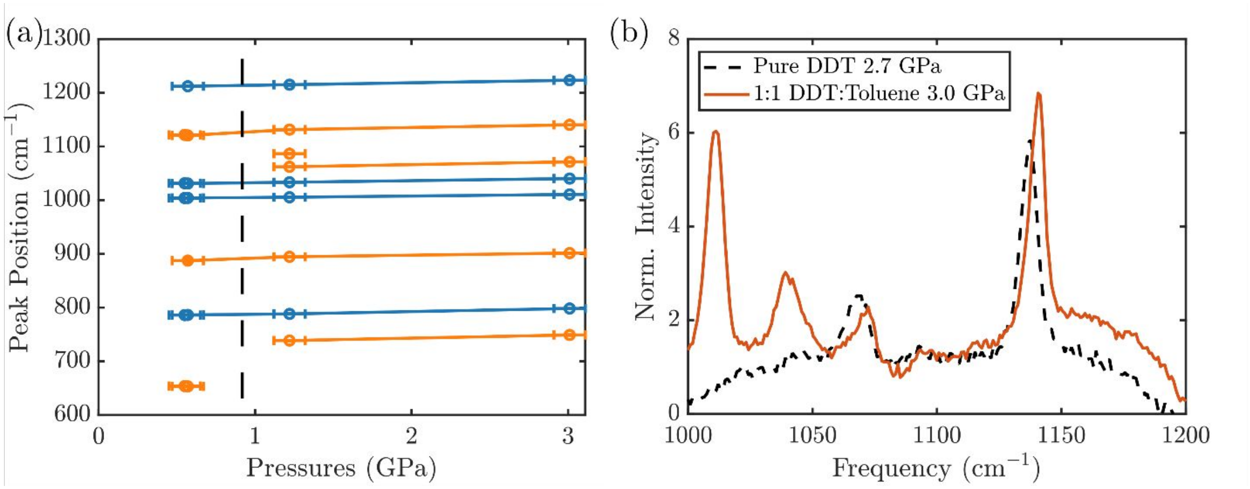

Figure S7. Characterization of Raman spectroscopy of 1:1 toluene:DDT mixture under pressure. (a) Peak positions of identified Raman vibrational modes versus pressure. Peaks identified as from DDT shown in orange, toluene in blue. (b) Comparison of Raman spectra of pure DDT (dashed black) and 1:1 toluene:DDT (solid red) at similar pressures near 3 GPa.

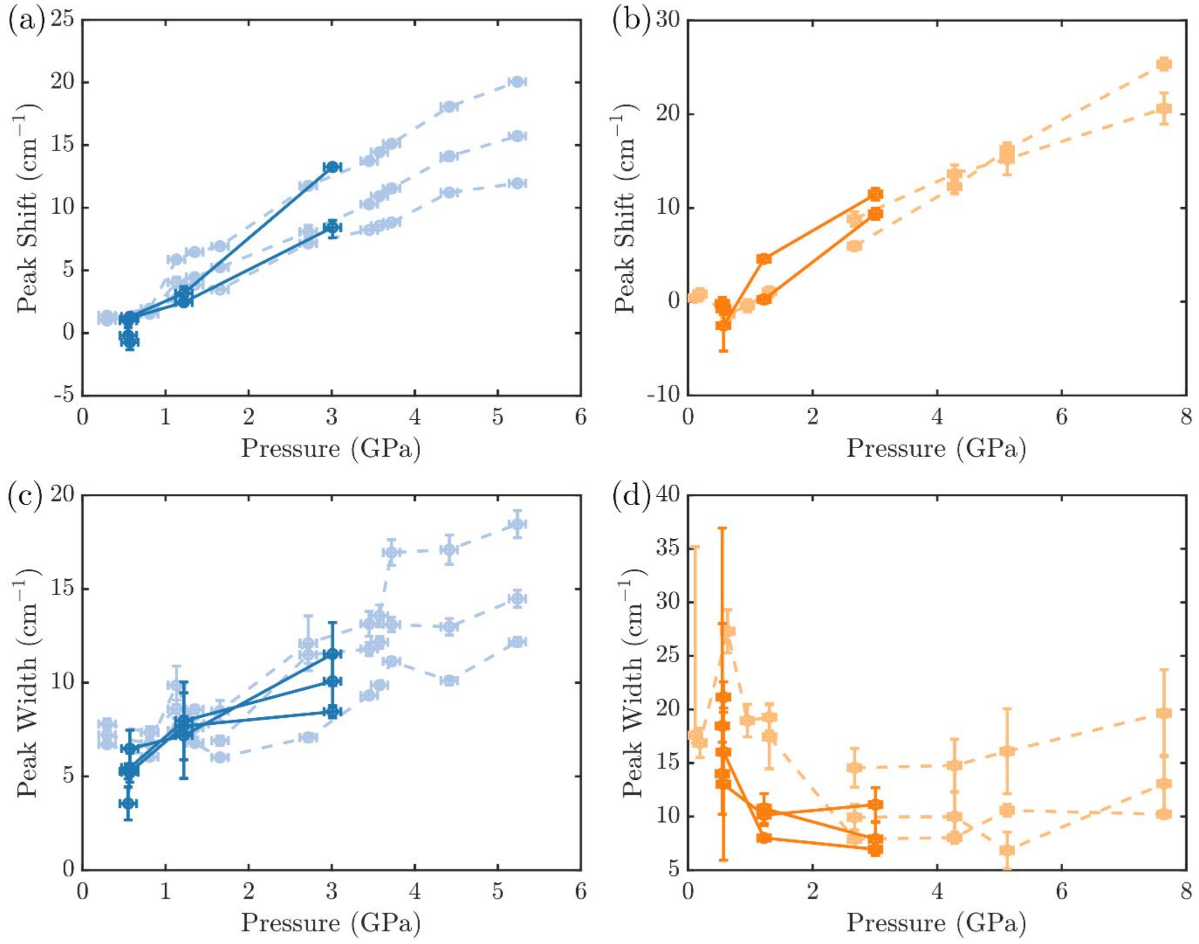

Figure S8. Characterization of Raman spectroscopy of 1:1 toluene:DDT mixture under pressure. (a) Raman peak shift of toluene vibrational modes in mixture (solid lines) compared to pure toluene (dashed lines) versus pressure. (b) Raman peak shift of 1-dodecanethiol vibrational modes in mixture (solid lines) compared to pure 1-dodecanethiol (dashed lines) versus pressure. (c) Widths of Raman peaks of toluene in the 1:1 toluene:DDT mixture versus pressure. (d) Widths of Raman peaks of DDT in the mixture versus pressure.

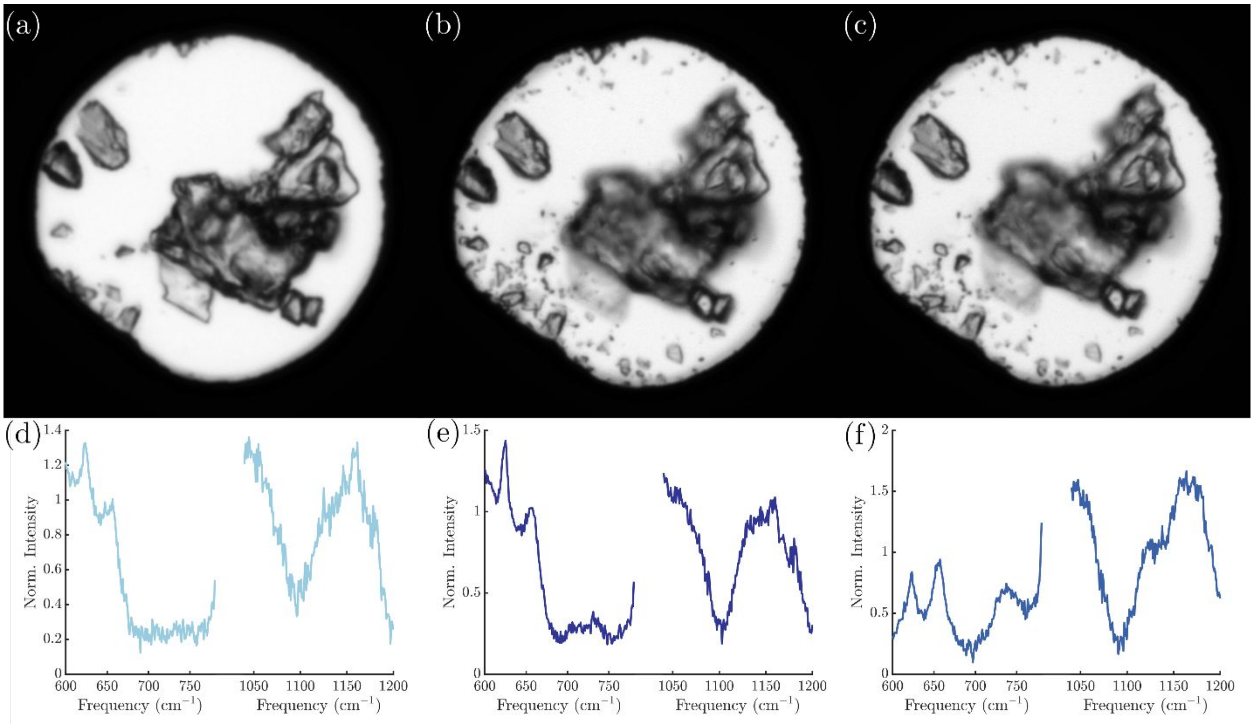

Figure S9. Comparison of Raman spectra with optical microscopy of 1:1 toluene:DDT under pressure. (a-c) Brightfield microscope images and (d-f) Raman spectra of the diamond anvil cell chamber (a and d) when initially loaded with 1:1 toluene:DDT at 1.0 GPa, (b and e) after holding at 0.8 GPa for 2 hours, and (c and f) after holding 0.9 GPa for 2 hours. Notice the increase in intensity in the ν(C-S)$_T$ peak from (d) to (e) to (f).
